# Experience or perception: What healthcare providers need when using the Utrecht Symptom Diary—4 Dimensional, a mixed-methods study

**DOI:** 10.1177/26323524241281748

**Published:** 2024-10-31

**Authors:** Tom Lormans, Everlien de Graaf, Carlo Leget, Saskia Teunissen

**Affiliations:** Julius Center for Health Sciences and Primary Care, University Medical Center Utrecht, Universiteitsweg 100, Utrecht 3584CG, The Netherlands; Julius Center for Health Sciences and Primary Care, University Medical Center Utrecht, Utrecht, The Netherlands; Care Ethics, University of Humanistic Studies, Utrecht, The Netherlands; Julius Center for Health Sciences and Primary Care, University Medical Center Utrecht, Utrecht, The Netherlands

**Keywords:** education, facilitators and barriers, implementation, palliative care, patient-reported outcome measures, reflection, sociality, spirituality, USD-4D

## Abstract

**Background:**

The Utrecht Symptom Diary—4 Dimensional (USD-4D), an adaptation of the Edmonton Symptom Assessment System, supports healthcare providers (HCPs) in identifying, monitoring, and exploring multidimensional symptoms and needs of patients in the palliative phase. For the USD-4D to be optimally implemented in clinical palliative care, it is essential to know and understand the needs of HCPs when using it.

**Objective:**

To identify and interpret the needs of HCPs when using the USD-4D in clinical palliative care, operationalized as perceived facilitators and barriers.

**Design:**

An explanatory mixed-methods study with a sequential design.

**Methods:**

Data were collected between October 2019 and September 2020. In phase I, quantitative data were collected through a survey targeting Dutch HCPs working in palliative care. Facilitators were identified as items answered positively by ⩾80% of participants, while barriers were identified as items answered negatively by ⩾20% of participants. In phase II, these identified facilitators and barriers were explored in depth through mixed composition focus groups. The Capability-Opportunity-Motivation-Behavior (COM-B) model was utilized to contextualize and interpret the perceived facilitators and barriers.

**Results:**

A total of 122 HCPs completed the survey, with 95% of the respondents being women with a mean age of 48 years and 72% being nurses. Additionally, 53% of the respondents had no prior experience with the USD-4D. In phase II, 21 HCPs participated in focus groups. 95% of the participants were women with a mean age of 49 years and 67% being nurses. HCPs pinpointed facilitators primarily related to the potential benefits of the USD-4D for daily patient care. Conversely, the identified barriers included issues related to HCPs’ behavior, knowledge gaps, uncertainty regarding their abilities and attitudes toward the USD-4D, and technical obstacles.

**Conclusion:**

Facilitators and barriers across all facets of the COM-B model were recognized, with a notable emphasis on motivational barriers. It should be acknowledged that facilitators and barriers can evolve throughout the implementation process, underscoring the importance of viewing implementation and integration as fluid and continuous endeavors. Facilitators and barriers are closely linked to HCPs’ reflective capacities, emphasizing the need for tailored intervention strategies that align with different stages of USD-4D implementation.

## Background

Patients with any life-limiting illness, that is, an illness that can reasonably be expected to end an individual’s life in the foreseeable future and has little or no prospect of cure,^1^ are faced with and experience multidimensional symptoms, issues, and needs, requiring patient-tailored care.^2^ Healthcare providers (HCPs) are tasked with relieving patients’ suffering and, in so doing, optimizing the quality of life of patients and their loved ones. Using patient-reported outcome measures (PROMs) in clinical practice supports HCPs in structurally assessing patients’ symptoms and needs in day-to-day multidimensional symptom management.

PROMs ideally are validated and standardized questionnaires, usually self-completed by patients to document their experiences and outcomes.^3^ These instruments play a vital role in guiding clinical decision-making, understanding patients’ needs, and evaluating the quality of care.^4^ In palliative care, PROMs have been tailored to measure outcomes that hold particular relevance and significance for patients in the palliative phase of their illness, thereby enhancing the overall quality of care provided. Despite the assumed positive impact of PROMs on healthcare,^5^ the challenges associated with implementing and utilizing these instruments have been widely acknowledged,^6–8^ a topic that will be explored further on in this introduction.

The Edmonton Symptom Assessment System (ESAS) is a widely and internationally recognized PROM with established feasibility in the assessment and monitoring of symptoms within clinical settings.^9,10^ The Utrecht Symptom Diary (USD) is a validated Dutch adaptation of the ESAS, specifically tailored to address prevalent physical and psychological symptoms encountered by patients in the palliative phase of their illness. Additional items were incorporated to monitor patients’ well-being and overall quality of life.^11^ The USD was developed to empower patients through self-assessment of their symptoms and needs. The adoption of PROMs, including the USD, is endorsed by the Netherlands Quality Framework for Palliative Care, emphasizing their value in enhancing patient-centered care.^12^

While HCPs across clinical palliative care settings and various Dutch palliative care networks have expressed the potential benefits of implementing multidimensional PROMs to enhance personalized palliative care, the existence of four-dimensional PROMs remained lacking. The development of The Utrecht Symptom Diary—4 Dimensional (USD-4D), detailed in Supplemental Appendix 1, filled this gap. To cover all four dimensions of palliative care, five expert-based items were added to the original USD. With the addition of these five items, the USD-4D consists of 21 items that are answered on an 11-point numerical intensity scale, allowing patients to articulate their symptoms’ severity (ranging from 0 for no symptoms to 10 for maximum intensity). Importantly, patients are encouraged to prioritize and highlight specific symptoms or needs for immediate attention, empowering their autonomy within the care process. Furthermore, the USD-4D promotes patient-centered dialogue concerning symptoms, needs, and preferences, facilitating a patient-driven approach to care.

The five socio-spiritual items were derived from a frequently used spiritual care instrument in Dutch palliative care: the Diamond Model (DM), an operationalization of the Ars Moriendi tradition. The theoretical foundation of the DM is available in Dutch, English, German, and Spanish.^13^ The DM is a validated hermeneutic instrument that has proven feasibility in supporting patients and HCPs in their dialogues on social and spiritual issues.^14,15^

**Figure 1.**
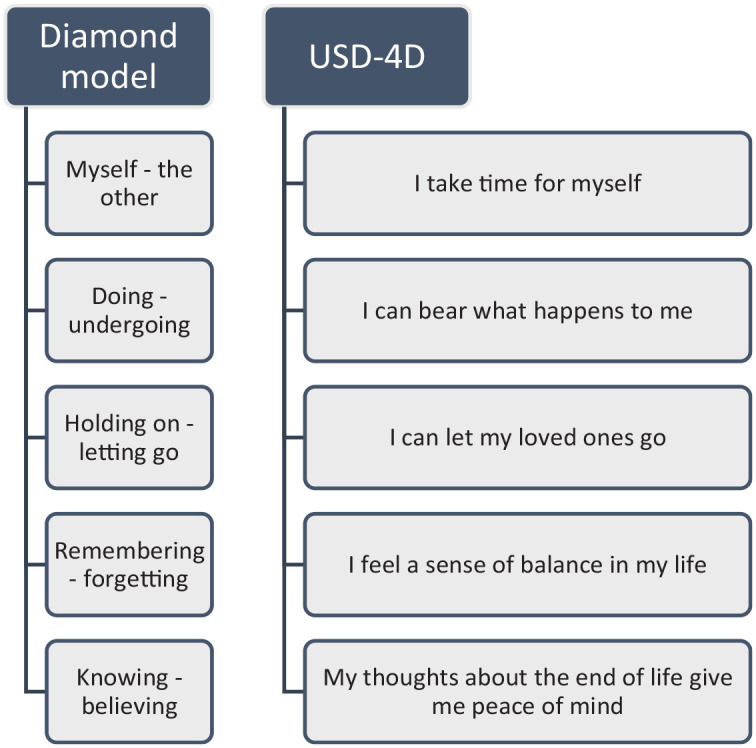
Anthropological polarities of the Diamond Model^13^ and their respective operationalized USD-4D items. USD-4D, Utrecht Symptom Diary—4 Dimensional.

Central to the DM is the concept of inner space: a metaphor that refers to a state of mind that enables an individual to relate to immediate emotions and attitudes evoked by any given situation. Five polarities can be used to reflect to what extent someone can experience inner space: myself versus the other, doing versus undergoing, holding on versus letting go, remembering versus forgetting, and knowing versus believing. For the USD-4D, these five polarities have been operationalized into five items, as shown in Figure 1. Depending on the patient’s point of view, these items reflect either the social or spiritual dimension or both.^16^ A study into the content validity of the USD-4D showed that patients perceive these socio-spiritual items to be comprehensive, relevant, and comprehensible.^17^

The USD-4D is mainly administered by nurses: they invite patients to use the USD-4D and do the initial exploration of symptoms and needs. Patient outcomes are then discussed within an interprofessional healthcare team so multidimensional care needs are adequately assessed. The USD-4D is both feasible for generalist and specialist palliative care, and its added value thrives in the interprofessional collaboration between these two. In the Netherlands, palliative care is provided by generalists and, when appropriate, in collaboration with HCPs specialized in palliative care. There is, however, no description of the required competencies or qualifications for these specialized HCPs. There are several educational programs that give HCPs the opportunity to educate themselves in palliative care.^12^ It was not until very recently that it was decided that palliative care should be structurally present in Dutch healthcare education.^18^

HCPs generally recognize the benefits of PROMs in both print and electronic formats, acknowledging their positive impact on patient care and their own professional responsibilities. The effective utilization of PROMs like the USD-4D enhances clinical practices by facilitating improved communication between patients and HCPs, empowering patient autonomy, and boosting patient satisfaction with healthcare services.^19–21^ However, the successful integration of PROMs into clinical settings poses various challenges, including time constraints, resource allocation, ensuring user-friendly interfaces for all stakeholders, and fostering HCPs’ willingness to incorporate PROMs into patient care.^21,22^ Even after implementation, HCPs may encounter obstacles in utilizing PROMs, such as concerns about patient vulnerability, limited knowledge and training, time management issues, technical complexities in using electronic PROMs, and the potential risk of dehumanizing patient care.^23,24^ Achieving successful integration and utilization of PROMs depends not only on the quality of the instrument itself but also on the readiness and willingness of end-users to embrace and effectively apply these instruments. Consequently, in implementing the USD-4D, it is important to consider and address the specific needs and concerns of HCPs to ensure its successful adoption and integration into clinical practice.

**Figure 2.**
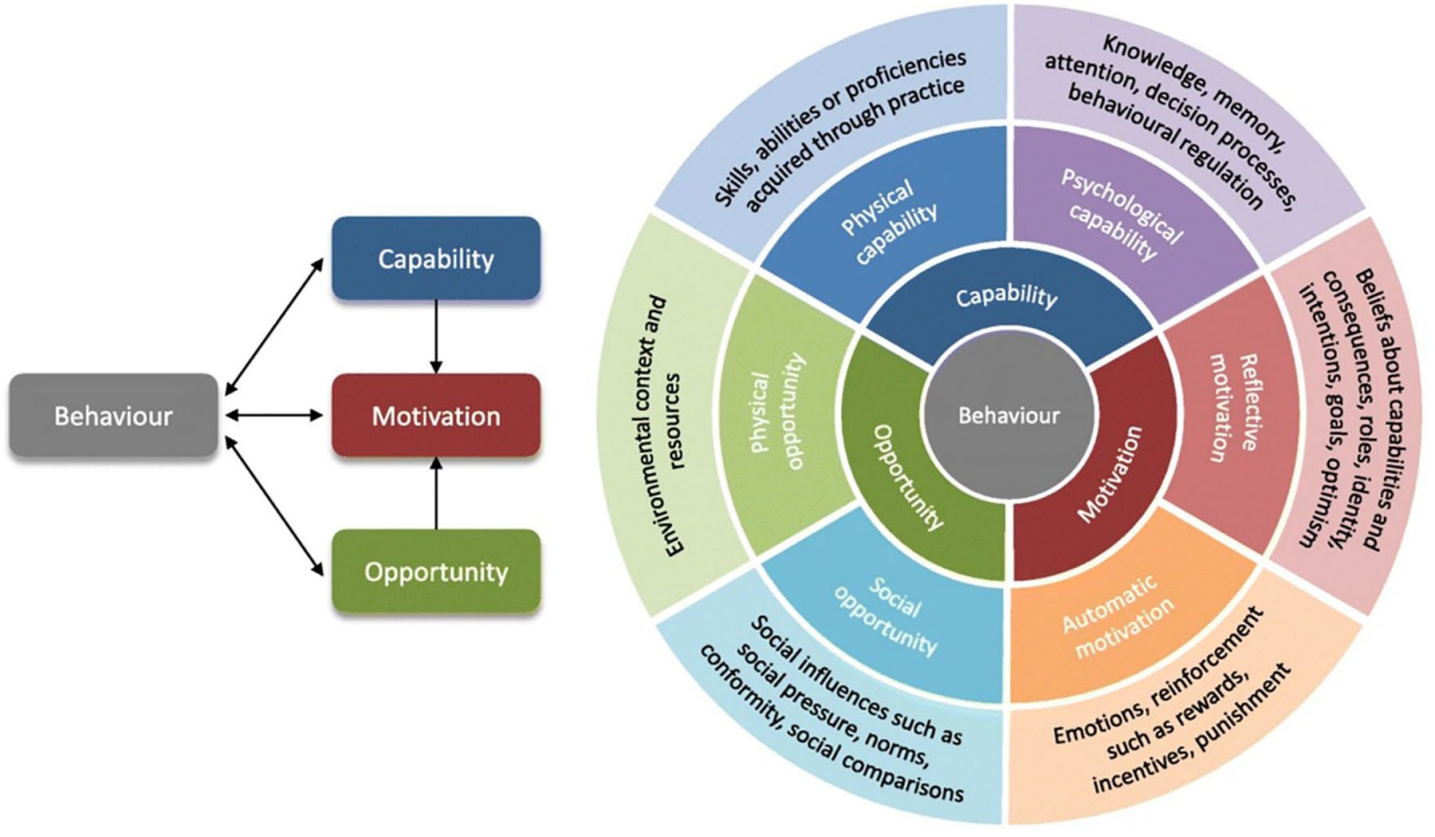
The COM-B model.^27^ COM-B, Capability-Opportunity-Motivation-Behavior.

While studies often stress technical requirements in PROMs implementation, it is equally important to consider HCPs attitudes and behaviors toward utilizing tools like the USD-4D when shaping implementation strategies.^25,26^ The Capability-Opportunity-Motivation-Behavior (COM-B) model proves to be a suitable framework for identifying facilitators and barriers related to HCPs’ attitudes and behaviors. This model, depicted in Figure 2, illustrates how behavior (B) results from the interplay of capability (C), opportunity (O), and motivation (M) factors.^27^

The aim of this study is to identify and interpret what HCPs need to use the USD-4D in clinical palliative care, operationalized through their perceived facilitators and barriers. Analyzed facilitators and barriers could then be used as input for developing adequate implementation and embedding strategies.^28^

## Methods

### Design

An explanatory mixed-methods study, employing a two-phased sequential quantitative-qualitative design, was conducted from August 2019 to September 2020.^29^ In the first phase, a quantitative approach was utilized, involving a cross-sectional survey to assess HCPs attitudes, experiences, and perceptions regarding the use of the USD-4D in clinical care. During the second phase, these survey results were explored in greater depth through focus groups (FGs). Both quantitative and qualitative data were considered equally important to the study’s objectives and were integrated during the data analysis phase to provide comprehensive insights.

For the quantitative phase of this report, we adhered to the STrengthening the Reporting of OBservational studies in Epidemiology (STROBE) statement.^30^ For the qualitative phase, we followed the Standards for Reporting Qualitative Research.^31^

### Population and context

This study targeted HCPs involved in palliative care, working across diverse healthcare settings including hospices, hospitals, home care, and nursing homes.

#### Phase I

For the quantitative phase, a convenience sample of mainly nurses working in palliative care was drawn. HCPs from all healthcare settings, including hospitals, home care, nursing homes, and hospices, were eligible to participate if they were ⩾18 years old and worked with patients suffering from life-limiting illnesses. Both generalists and specialists were included. Generalists work within an organization where palliative care is part of the whole, but does not predominate, for example, palliative care unit in hospital. Specialists work within an organization where all patients receive palliative care, for example, hospice care. Recognizing that barriers to using the USD-4D might differ between HCPs with (HCPs-e) and without clinical experience (HCPs-ne) in using this PROM, prior experience with the USD-4D was not an eligibility criterion.

#### Phase II

For the qualitative phase of this study, a purposive sample of HCPs was drawn to attempt to ascertain a representative sample of all relevant healthcare disciplines that use the USD-4D in clinical care: nurses, general practitioners, social workers, and chaplains. Eligibility criteria were equal to those of phase I. Researchers asked regional network coordinators to contact HCPs with information about the study. Interested HCPs received detailed study information and an invitation from the researcher (TL) to participate in a FG. Acceptance of the invitation implied consent to use their data for research purposes.

### Outcome

#### Phase I

The primary outcome was the identification of HCPs’ perceived facilitators and barriers to using the USD-4D in clinical care. Facilitators were defined as items positively answered by ⩾80% of participants. Barriers were defined as items negatively answered by ⩾20% of participants.

#### Phase II

This phase aimed to provide an explanation of the facilitators and barriers identified through the survey to better understand these results and identify underlying mechanisms.

### Data collection

#### Phase I

A 49-item survey was purposefully designed to identify facilitators and barriers when using the USD-4D in clinical palliative care. The survey reflected how the USD-4D was used in clinical practice and consisted of four sections: introducing the USD-4D, functioning of the USD-4D, using the USD-4D in a multiprofessional healthcare team, and competencies and introsprection concerning the USD-4D (see Supplemental Appendix 1).

Responses were recorded on a four-point Likert scale (1 being “totally disagree” and 4 being “totally agree”). The research team, experts in multidimensional symptom management and social and spiritual care, as well as a local council of nurse practitioners (*n* = 2) and patients (*n* = 2) established the face validity of the survey by assessing the relevance and suitability of the survey items for measuring the desired outcomes.^32^

Data were collected during two palliative care educational meetings in October 2019. During these meetings, HCPs attended a presentation by TL, with a background in chaplaincy, and EdG, an experienced qualitative researcher with a nursing background, about the use of the USD-4D in clinical palliative care. Participants practiced using the PROM, ensuring that both experienced and inexperienced HCPs had a common understanding of the USD-4D and its clinical context. After these sessions, HCPs completed the survey anonymously on paper, taking approximately 15 min.

#### Phase II

Data were collected through FGs held from July to September 2020. FGs were suitable for discussing and explaining survey results among HCPs. Due to COVID-19 regulations, these sessions were conducted online via ZOOM. FGs lasted between 90 and 120 minutes and were digitally recorded using the ZOOM software^33^ (both video and audio). TL and EdG facilitated the FGs.

During the FGs, facilitators and barriers identified in the survey were explored in depth. A topic guide, primarily based on the survey outcomes and existing literature, was used. Researchers presented the survey results to participants and highlighted key issues for discussion. HCPs were then invited to share their perceptions, thoughts, and ideas. Data collection continued until thematic saturation was achieved using an inductive thematic coding approach.^34^

### Data analysis

#### Phase I

Survey data were transposed to the IBM Statistics Package for Social Sciences (SPSS) version 25 and analyzed using descriptive statistics.^35^ Survey responses were dichotomized to focus on positive and negative reactions. The COM-B model, defined in Table 1, served as the analytical framework.

**Table 1.** Definition of the domains of the COM-B model.^27^

COM-B model	Explanation
Capability	Refers to knowledge, skills, and abilities: an individual’s capability to modify behavior
Opportunity	Refers to external factors in the environment that influence an individual’s behavior
Motivation	Refers to internal processes that concern the willingness to change
Behavior	Refers to the behavior of an individual

COM-B, Capability-Opportunity-Motivation-Behavior.

#### Phase II

Phase II FG discussions were transcribed verbatim and analyzed iteratively using thematic analysis. The domains of the COM-B model served as a framework for categorizing the data. TL and EdG discussed and assigned codes to the data, mapping these codes to the relevant COM-B domains. Continuously cycling between the data and the COM-B model domains allowed for an optimal response to the research question. Peer debriefing sessions were conducted to reflect on the findings, and any differences in perception were discussed by the research team (TL, EdG, CL, ST). NVivo 12 software was utilized for data analysis.^36^

#### Trustworthiness

To ensure trustworthiness, FGs were video- and audio-recorded, transcribed verbatim, and field notes were taken immediately after each FG session. The FG interviews were discussed and analyzed by two researchers (TL and EdG) to enhance credibility. Any discrepancies were addressed and resolved in discussions with a multiprofessional research team until consensus was reached. Member checks were utilized during the FGs to verify and correct any mistakes in interpretation.^37^

### Ethical approval

The study was conducted in compliance with the principles of Good Clinical Practice and in accordance with the principles of the Declaration of Helsinki^38^ and the General Data Protection Regulation.^39^ As a part of the INZICHT project, the Medical Research Ethics Committee Utrecht confirmed that the Medical Research Involving Human Subjects Act did not apply to this study (reference number 19-602/C, 2019).

## Results

A total of 122 participants completed the survey, with a mean age of 48 years, predominantly women (95%), and mainly nurses (72%). Forty-seven percent of the HCPs had prior experience with the USD-4D, with those experienced users more likely to be nurses working in hospices. Additionally, 21 HCPs participated in the FGs, 95% of whom were women with a mean age of 49 years, and the majority were nurses (67%).

Detailed characteristics of these participants are presented in Table 2.

**Table 2.** Participants’ characteristics.

Characteristics	Survey		Focus groups
	No experience with USD-4D	Experience with USD-4D	
Number of participants	65	57	21
Gender, *N* (%)			
Female	64 (99)	52 (91)	20 (95)
Age, mean years	48	48	49
Philosophy of life, *N* (%)			
None	3 (5)	2 (4)	4 (19)
Catholic	1 (2)	7 (12)	1 (5)
Protestant	57 (88)	45 (79)	16 (76)
Atheist	2 (3)	1 (2)	0 (0)
Other	2 (3)	2 (4)	0 (0)
Practicing religion, *N* (%)			
Yes	42 (65)	51 (89)	14 (67)
Occupation, *N* (%)			
Healthcare assistant	22 (37)	3 (5)	0 (0)
Nurse	38 (58)	49 (86)	14 (67)
General practitioner	0 (0)	0 (0)	1 (5)
Social worker	0 (0)	0 (0)	1 (5)
Chaplain	4 (6)	3 (5)	4 (19)
Manager	0 (0)	1 (2)	1 (5)
Paramedic	1 (2)	1 (2)	0 (0)
Experience, mean years	24	26	23
Palliative care, *N* (%)			
No	5 (8)	0 (0)	0 (0)
Yes, generalist^a^	53 (82)	22 (39)	18 (86)
Yes, specialist^b^	7 (11)	35 (61)	3 (14)
Palliative care education, *N* (%)			
None	10 (15)	4 (7)	0 (0)
Yes, training within basic education	4 (6)	6 (11)	11 (52)
Yes, one time training	45 (69)	28 (49)	2 (10)
Yes, periodical training	1 (2)	17 (30)	3 (14)
Yes, post-higher vocational education for registered nurses	5 (8)	2 (4)	5 (24)
Setting, *N* (%)			
Home care	40 (62)	11 (19)	6 (29)
Hospital	4 (6)	2 (4)	3 (14)
Nursing home	13 (20)	2 (4)	0 (0)
Hospice	8 (12)	42 (74)	12 (57)

a Working within an organization where palliative care is part of the whole, but does not predominate, for example, palliative care unit in hospital.

b Working within an organization where all patients receive palliative care, for example, hospice care.

USD-4D, Utrecht Symptom Diary—4 Dimensional.

**Figure 3.**
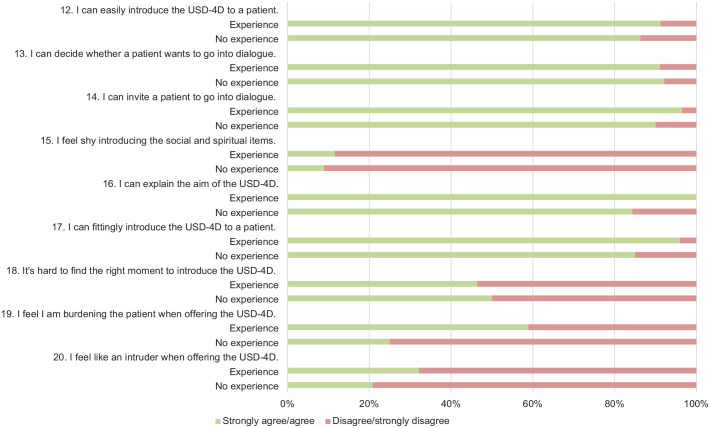
Introducing the USD-4D. USD-4D, Utrecht Symptom Diary—4 Dimensional.

**Figure 4.**
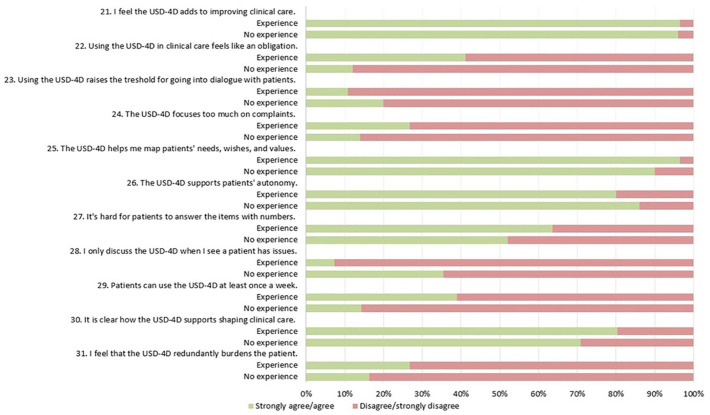
Functioning of The USD-4D. USD-4D, Utrecht Symptom Diary—4 Dimensional.

**Figure 5.**
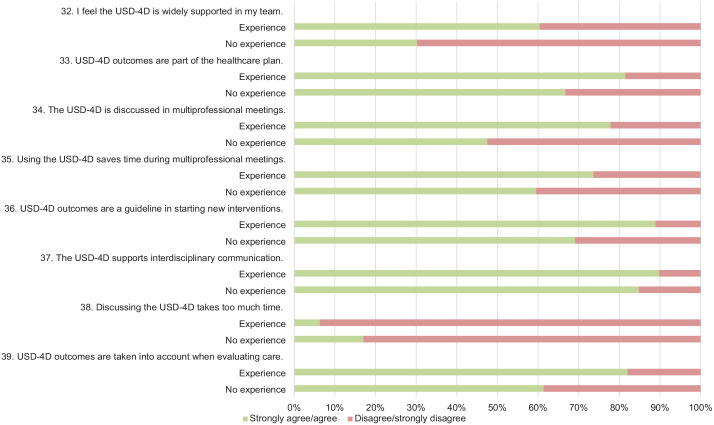
Using the USD-4D in a multiprofessional healthcare team. USD-4D, Utrecht Symptom Diary—4 Dimensional.

**Figure 6.**
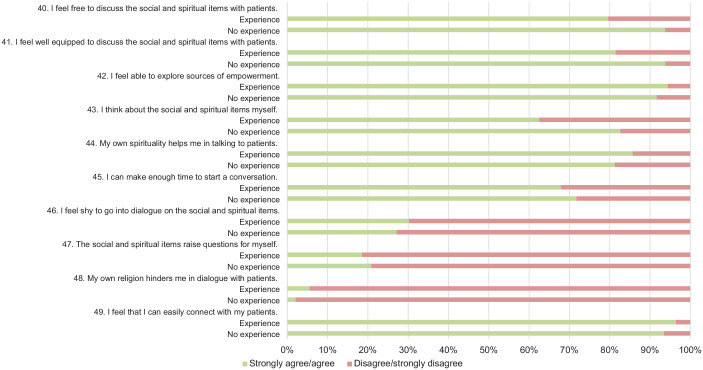
Competencies and introspection concerning the USD-4D. USD-4D, Utrecht Symptom Diary—4 Dimensional.

Survey results are illustrated in Figures 3–6. Results are presented generally for all HCPs; however, when differences between HCPs with (HCPs-e) and without prior experience (HCPs-ne) with the USD-4D are observed, these distinctions are described accordingly.

## Facilitators and barriers

### Capability

#### Skills and abilities

##### Facilitators

In phase I, HCPs reported a high level of confidence in their ability to work with the USD-4D. Specifically, 93% stated they felt capable of introducing the USD-4D, 93% could invite patients to engage in dialogue about the USD-4D, and 90% could do so in a manner that suited patients’ needs. Additionally, 94% perceived that they were able to connect with patients effectively and 93% indicated that they could explore sources of empowerment through the USD-4D. Furthermore, 88% of HCPs felt confident in discussing social and spiritual issues with patients. In phase II, FG discussions reinforced these findings. Participants affirmed that the aforementioned capabilities facilitated their use of the USD-4D.

##### Barriers

In phase I, it became evident that 42% of HCPs found it challenging to determine the right moment to introduce the USD-4D to patients. In phase II, participants expressed a critical perspective on the facilitators identified through the survey results. It was observed that HCPs often tend to overestimate their skills and abilities in using the USD-4D in clinical practice. A social worker noted, “You think it’s all easy, but then you step into someone’s room and you find yourself left speechless more than once.” The discussions revealed that HCPs do indeed face difficulties in introducing the USD-4D and inviting patients to engage in dialogue, particularly concerning the socio-spiritual items. A nurse commented, “Although it sometimes appears to be difficult to invite patients to talk about these issues, practice makes perfect. You just need to keep trying.”

#### Knowledge and awareness

##### Facilitators

In phase I, HCPs reported high confidence in their ability to explain the purpose of the USD-4D (92%) and to assess whether a patient could utilize the tool (92%). The latter was discussed extensively in phase 2 as a barrier too.

##### Barriers

In phase I, 29% HCPs felt that the USD-4D items were unclear. Of HCPs-e, 39% found it unclear how the USD-4D supports clinical care. Additionally, 21% of HCPs-ne reported that the USD-4D items raised too many questions, making them unclear.

In phase II, FG participants emphasized that not all colleagues had a clear understanding of the USD-4D’s purpose. They also noted that HCPs generally lacked knowledge about the social and spiritual issues addressed by the USD-4D items. Furthermore, participants observed significant variability in how the USD-4D is applied in clinical practice. As one nurse remarked, “I see that some colleagues use the items as a guideline for conversations and others present the literal items to patients. Both can be used in my opinion, but there is no consensus.” Furthermore, FG participants highlighted a significant concern considering HCPs assessing whether patients can use the USD-4D: the potential for gatekeeping. One nurse noted, “It comes down to experience. As you gain more experience, you learn to determine which patients are open to using the USD-4D and which patients are not.” Another nurse added: “… This leads to not asking patients whether they want to use the USD-4D. All around me, I actually hear from everyone that it is pre-determined which patients can and cannot use the USD-4D.”

### Opportunity

#### Resources and time

##### Facilitators

In phase I, 89% of HCPs noted that discussing USD-4D outcomes during multidisciplinary consultations did not consume excessive time.

In phase II, HCPs highlighted the importance of technical systems that streamlined the use of the USD-4D in clinical practice. A nurse emphasized the benefit of digitally presenting USD-4D outcomes during multiprofessional meetings: “During our multiprofessional meetings, USD-4D outcomes are presented digitally so everyone can see them when we discuss a patient’s symptoms and needs. These outcomes are presented as graphs, so it is clear at a glance how outcomes vary over time.” Utilizing a digital application was seen to enhance patient convenience and aid HCPs in interpreting data seamlessly within healthcare plans. A physician mentioned the efficiency gained: “It is more convenient when patients fill in the USD-4D through an application and the results are immediately brought together.”

##### Barriers

In phase 1, HCPs expressed challenges in finding sufficient time to engage in meaningful dialogues with patients about the outcomes (30%).

This was elaborated on in phase II by a nurse: “I try to ask some more questions when I see my patients, but usually I do not have enough time to go and sit with them.” Additionally, HCPs indicated that some organizations lack access to digital applications and support systems, hindering optimal utilization of the USD-4D in clinical practice.

#### Norms and conformity

##### Facilitators

In phase I, HCPs recognized that the USD-4D enhanced interdisciplinary communication (88%).

In phase II, nurses emphasized the importance of the USD-4D fostering interdisciplinary collaboration within the team, not only benefiting expert HCPs such as social workers and chaplains.

##### Barriers

In phase I, it was evident that HCPs perceived a lack of widespread support for the USD-4D within their multiprofessional teams (55%), with the tool not being consistently discussed during collaborative meetings (36%). Moreover, some HCPs felt that offering the USD-4D weekly was too frequent (27%).

In phase II, FG participants unanimously stressed the necessity of robust multiprofessional team support for successful USD-4D integration. Varied frequencies of USD-4D utilization among different care settings were noted. A nurse explained: “We offer the USD-4D once every two weeks to patients and the USD every week.” Another nurse told: “We try to use the USD-4D every Sunday.” Furthermore, the absence of colleague support dissuaded HCPs from using the USD-4D, with a participant highlighting how a single caregiver’s stance could significantly impact the tool’s adoption within a whole setting.

### Motivation

#### Emotions and incentives

##### Facilitators

In phase I, HCPs emphasized that the USD-4D plays an important role in mapping patients’ symptoms, needs, and wishes (93%) and facilitating multiprofessional communication (88%). Additionally, HCPs recognized the USD-4D as a tool that improves clinical care, with 96% acknowledging its positive impact.

In phase II, it became evident that the USD-4D significantly contributes to patient-centered palliative care, serving as a key motivation for HCPs to utilize the PROM in clinical practice. A nurse expressed: “I personally think it is a good instrument because some themes just are not brought to attention easily. By using the USD-4D once a week, themes and issues emerge that would otherwise remain unexplored.”

##### Barriers

In phase I, HCPs-e highlighted various barriers related to the incentives for utilizing the USD-4D in clinical practice. These barriers included concerns that introducing the USD-4D could burden patients (57%), using the USD-4D redundantly burdened patients (27%), the PROM not adequately supports patients’ autonomy (20%), and HCPs felt a sense of obligation in using the USD-4D rather than wanting to use it (41%). Moreover, HCPs-e also felt that the USD-4D excessively focused on complaints rather than sources of empowerment (27%).

HCPs-ne perceived that USD-4D outcomes were not integrated into the care plan (33%) and would only discuss the USD-4D when patients raised concerns (35%). Additionally, they found it challenging to initiate dialogues with patients using the USD-4D, resulting in a raised threshold for engagement (20%). HCPs-ne also noted that using the USD-4D did not streamline multiprofessional meetings (33%) and perceived it to be difficult for patients to answer the USD-4D items on a numeric scale (58%).

In phase II, HCPs-e suggested that using the USD-4D could enhance efficiency in multiprofessional meetings by enabling patients to prioritize their needs.

Moreover, participants expressed concerns about providing optimal care without burdening patients excessively, leading some HCPs to forgo asking patients to complete the USD-4D. However, a nurse added: “I think that patients are very able to tell us when they do not feel like answering or discussing the socio-spiritual items. And we should trust that.” Another nurse added: “It is a bit worrisome that people feel so burdened by it, because then you will probably quit or skip items that could matter on short notice. . . . In clinical care, I have noticed that there is a growing resistance towards lists. This could possibly be an explanation for the numbers you see here: that HCPs think ‘now I have to go and ask all these questions again,’ and that that gives rise to a certain reticence.” Another nurse experienced this as “. . . another ‘list’ I have to present to patients before having a conversation. I don’t always want to use it.”

#### Beliefs about capabilities and consequences

##### Facilitators

In phase I, HCPs reported feeling comfortable introducing the socio-spiritual items to patients (89%). Moreover, they expressed that their personal spirituality aided them in discussing these topics (84%) and that their own religious beliefs did not impede dialogue (96%).

In phase II FGs, nearly all participants mentioned that increased confidence among HCPs resulted in greater satisfaction with using the USD-4D.

##### Barriers

In phase I, HCPs expressed hesitancy in discussing social and spiritual matters (29%). HCPs-e reported feeling intrusive when introducing the USD-4D (31%).

In phase II, it became apparent that there was little to no emphasis placed on engaging in conversations about social and spiritual themes. A physician stated: “During my studies, no attention was paid to how to discuss a subject like death with patients. Even now, I notice that I am sometimes shy and don’t know what questions to ask.” FG participants added that HCPs tended to prioritize having a “good conversation” when addressing social and spiritual matters, setting a high standard for actually initiating dialogue. A nurse pointed out: “people tend to focus on wanting to have a good, deep, and meaningful conversation, thus putting a lot of pressure on it. Instead, why not just go and talk to patients, which makes it more accessible?” Moreover, it was observed that not all HCPs had the same approach to using the USD-4D: nurses viewed it as a tool for signaling and monitoring, while chaplains saw it more as a referral tool. A chaplain mentioned: “I educate nurses in my team to use the USD-4D more frequently, so I get referred to patients more quickly.” FG participants highlighted that HCPs sometimes viewed the social and spiritual aspects as the sole responsibility of social workers and chaplains, perceiving these dimensions as beyond the nursing scope.

## Discussion

The aim of this study was to identify what HCPs need when using the USD-4D in palliative care. These needs were operationalized through the COM-B model as perceived facilitators and barriers to using the USD-4D in clinical care in terms of capability, opportunity, and motivation. The results of this study indicate that both facilitators and barriers were identified for all domains of the COM-B model. Overall, the findings show that HCPs, mainly nurses, perceive the use of the USD-4D as improving multidimensional, patient-centered palliative care. Common barriers, which are in line with the findings from other studies, included insufficient time, lack of knowledge, and inadequate technological infrastructure.^26,27,38^ This study adds that most barriers were identified in the motivation domain of the COM-B model. Another study that used a behavior model to examine the facilitators and barriers toward PROMs also found that most barriers were related to users’ motivation.^40^ Aside from the perceived uselessness of a PROM, other studies concerning the implementation of PROMs do not focus as much on the motivation of users.^41^

As the COM-B model intends to show, categorization of the facilitators and barriers through this model demonstrated that capability, opportunity, and motivation interact in HCPs’ behavior. For example, HCPs who lack training (physical capability) and knowledge regarding the USD-4D (psychological capability) were less self-assured in using the PROM (reflective motivation). Since this model was used to contextualize the findings, the identified facilitators and barriers concern HCPs’ attitudes and behavior toward the instrument and the context in which it is used, which differs from many other studies.^42–45^

Thus indicating facilitators and barriers through a behavior model like the COM-B model demonstrates that these factors are not absolute but rather dynamically involved. This becomes even more apparent when the identified facilitators and barriers are contextualized using both the COM-B model and learning frameworks.

**Figure 7.**
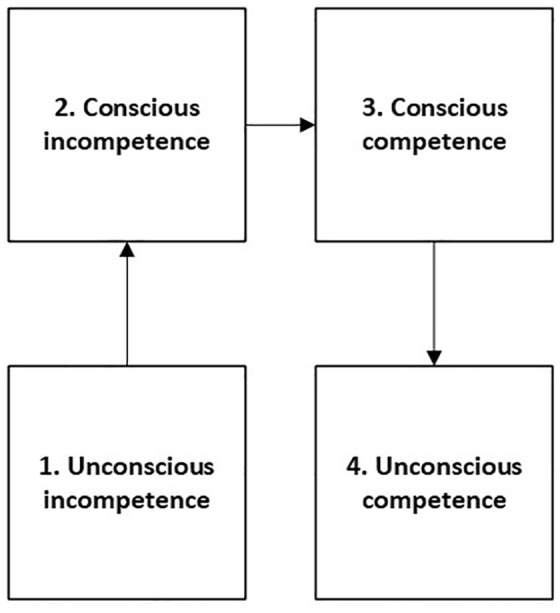
The conscious competency learning model.

In medical and healthcare education, the conscious competence learning model is the most referenced learning framework. This model (Figure 7) originates from the coaching industry and illustrates how people progress through four stages of competence: unconscious incompetence, conscious incompetence, conscious competence, and unconscious competence.^46^

**Figure 8.**
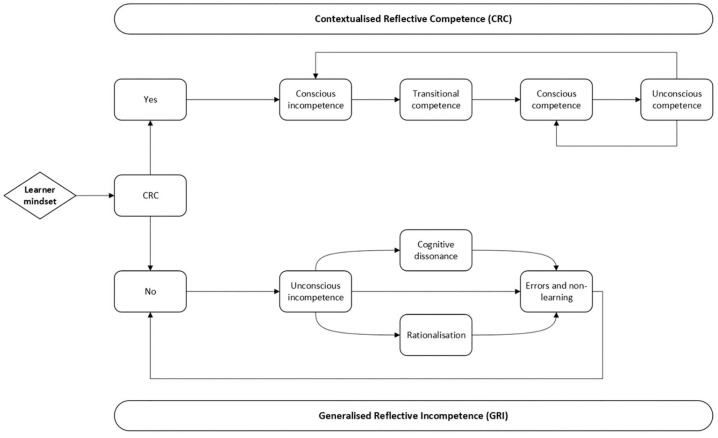
Learning model for “contextualized reflective competence.”

A recent study has highlighted that the lack of ongoing reflective practice is a significant flaw in this framework: reflective practice should continue once a skill has been learned (i.e., unconscious competence). Therefore, the conscious competence learning model has been adapted into a framework for Contextualized Reflective Competence (CRC). In this framework, mastery is not equivalent to unconscious competence; rather, it is defined as possessing reflective competence: having the ability to identify unconscious incompetence in yourself as well as others.^47^ Figure 8 depicts the CRC framework.

When HCPs appropriately reflect, they move through the upper loop of the framework; when they do not possess this skill, they move through the lower loop. The lower loop is a state of temporary reflective incompetence, resulting in errors and a failure to learn from them. The upper loop demonstrates how competence is developed. It is crucial for individuals to operate within a context of reflective competence, as this context enables them to remain in the upper part of the diagram. Developing overconfidence—making mistakes without reflecting on them and thus not learn from them—can cause competent individuals to move to the lower part of the diagram.

According to the CRC framework, experiencing facilitators or barriers to using the USD-4D in terms of capabilities, opportunities, and motivation depends on HCPs’ reflective capabilities, as well as their experience and willingness to learn, continue learning, and retain learning. Early career HCPs are generally more open to recognizing that they have much to learn, whereas mid-career HCPs might believe that they cannot learn anything new.

HCPs who are unconsciously incompetent in using the USD-4D do not experience any barriers to its usage, as they are simply unaware of them. They do not know whether they are doing something right or wrong and do not recognize facilitators or barriers. Learning about the USD-4D, and consequently recognizing one’s needs in terms of capabilities, opportunities, and motivation, begins when HCPs are consciously incompetent and are willing to learn from mistakes they will make anyway. HCPs who are aware of the challenges they face due to lack of education or experience can work to improve their skills.

As HCPs further develop their skills and more frequently use a PROM like the USD-4D, they encounter facilitators and barriers. HCPs will have to accept their mistakes while they acknowledge their successes as well. Making mistakes does not mean you are doing everything wrong. During this stage, which the authors of the CRC call the stage of transitional competence, there is a risk of developing overconfidence.^47^ This may be particularly evident when considering the positive attitude of HCPs toward spiritual care in this study. HCPs indicate that they feel comfortable introducing socio-spiritual items, discussing them, and exploring sources of empowerment, even though these aspects are often viewed as challenging.

Eventually, HCPs will consistently get things right and master the use of the USD-4D. However, even at this stage, mistakes can still occur. This is particularly true for HCPs who feel confident in determining whether patients are able to use the USD-4D. These HCPs may stop reflecting on their actions and, as a result, stop learning. Frequently, HCPs believe that the USD-4D redundantly burdens patients, potentially leading to what is known as “gate-keeping.”^48^ This can be seen as a form of cognitive dissonance: HCPs believe the USD-4D supports clinical palliative care but simultaneously do not use it because they think it burdens patients. However, a recent study showed that patients appreciated being invited to complete the USD-4D and discuss the items.^49^ Additionally, some HCPs-e argued that introducing the socio-spiritual items can do more harm than good, believing that omitting these items is preferable, while in reality, HCPs feel uncomfortable asking these questions. This can refer to what literature calls a rationalization of a moral situation, where HCPs reinterpret the consequences of their actions in a more positive light.^50,51^

In line with previous research, this study shows that HCPs have educational needs when it comes to adequately using the USD-4D in clinical care. Implementation strategies should therefore be developed to transfer knowledge effectively. Education should focus on HCPs’ reflective capabilities and their actual needs within the context in which the USD-4D is being used.

Training and coaching-on-the-job are suitable strategies for addressing barriers related to HCPs’ perceived lack of skills in using the USD-4D and going into dialogue about issues concerning the social and spiritual dimensions of palliative care. HCPs maintain high standards for having a “good conversation,” which is reflected in the demanding skillset that is supposedly required.^52,53^ This only raises the threshold for even starting a conversation. Practice opportunities, such as conversation-training with colleagues or, ideally, actors, will increase both HCPs’ skills and confidence.^54^

Training and collective reflection can also make HCPs aware of how introducing the USD-4D to a patient is perceived.^17,47^ Furthermore, instead of monopolizing dialogues with patients on the social and spiritual dimensions, expert HCPs such as social workers and chaplains should contribute to normalizing these dialogues by coaching and empowering their colleagues on the job. Ongoing support will facilitate the use of the USD-4D.

## Strengths and limitations

A strength of this study was the combined quantitative and qualitative approach, which allowed for an in-depth explanation of the current experiences and subsequent perceived facilitators and barriers of HCPs when using the USD-4D. Another strength of this study is that it specifically focuses on what HCPs require when using the USD-4D. Since the study results align with prior research, the findings suggest that, although presented in an adapted formulation, these insights can be used to evaluate HCPs’ needs when using multidimensional PROMs in other contexts as well.

A limitation of this study was that it is conducted in the center of the Netherlands. Conducting the study in this specific geographic area resulted in a higher proportion of HCPs with religious involvement (⩾42%) compared to national averages. The study area, known as the Dutch “Bible Belt,” is recognized for its relatively strong religious presence. While hospices in this region tend to have a more pronounced religious affiliation, it is not excessively strict. Subgroup analysis revealed no significant differences in outcomes between religiously practicing and non-practicing HCPs, so results ought to be generalizable.

Another limitation of the study was the uneven distribution of HCPs, presumably due to the focus on the social and spiritual dimensions of palliative care. Consequently, the study population was predominantly composed of nurses and chaplains. The inclusion of general practitioners on the one hand and social workers on the other hand proved to be more challenging. The latter possibly has to do with the organization of the profession. Within FGs, only one physician and one social worker participated. Despite the underrepresentation of these professions in the study, it accurately mirrors clinical practice, where nurses primarily engage with the USD-4D. Additionally, the underrepresentation of participants from hospital settings poses challenges to the generalizability of the results to this specific setting.

Considering the COVID-19 regulations, the study necessitated online FGs instead of in-person meetings. This adaptation made organizing and participating in the FGs more convenient and efficient. Online discussions facilitated a more focused working environment with fewer distractions.^55^ However, the smaller group size in online sessions might have impacted group dynamics, potentially limiting discussions and posing challenges for researchers who had to actively engage participants by asking more questions. Additionally, technical issues and unreliable internet connections at times hindered smooth communication among participants.

### Recommendations for clinical practice

The primary recommendation for clinical practice is simply for HCPs to start using the USD-4D. Using the USD-4D affects HCPs’ skills, behavior, and attitude. Implementation and embedding strategies should target both the behavior and attitude of HCPs, along with integrating the USD-4D structurally and technically into clinical care. Strategies addressing behavioral and technical obstacles need to be devised. Support for HCPs in using this PROM is essential to ensure optimal patient benefits. Education should enhance HCPs’ reflective abilities and offer practical guidance on incorporating the USD-4D in clinical settings. Additionally, promoting interprofessional collaboration among healthcare teams can encourage professionals to engage with the USD-4D. Social workers and healthcare chaplains play crucial roles in assisting generalists in evaluating the social and spiritual dimensions of palliative care.

### Recommendations for future research

Future research should concentrate on establishing the best practices for implementing the USD-4D. A starting point would involve examining the essential preconditions for building trust in utilizing this PROM and understanding its implications for patients. Subsequently, investigating the impact of these implementation strategies on adoption and integration, as well as assessing how the implementation of the USD-4D influences regular palliative care procedures, would be essential. Furthermore, research should explore how the USD-4D can effectively guide necessary care.

## Conclusion

HCPs, particularly nurses, both identify and experience facilitators and barriers when using the USD-4D in clinical palliative care. The application of the COM-B model has revealed that HCPs perceive the USD-4D as beneficial for multidimensional patient-centered care, with most barriers being interpreted as motivational barriers. The experience of these facilitators and barriers is contingent upon HCPs’ reflective competencies. Additionally, facilitators and barriers may evolve during the implementation phase, emphasizing the importance of viewing implementation and integration as continual processes. Consequently, intervention strategies should be customized to the different phases of USD-4D implementation. HCPs need to be educated in reflective skills to enable them to reflect on their actual needs and the practical aspects of using the USD-4D in palliative care.

## Supplemental Material

sj-docx-1-pcr-10.1177_26323524241281748 – Supplemental material for Experience or perception: What healthcare providers need when using the Utrecht Symptom Diary—4 Dimensional, a mixed-methods study Supplemental material, sj-docx-1-pcr-10.1177_26323524241281748 for Experience or perception: What healthcare providers need when using the Utrecht Symptom Diary—4 Dimensional, a mixed-methods study by Tom Lormans, Everlien de Graaf, Carlo Leget and Saskia Teunissen in Palliative Care and Social Practice

sj-docx-2-pcr-10.1177_26323524241281748 – Supplemental material for Experience or perception: What healthcare providers need when using the Utrecht Symptom Diary—4 Dimensional, a mixed-methods study Supplemental material, sj-docx-2-pcr-10.1177_26323524241281748 for Experience or perception: What healthcare providers need when using the Utrecht Symptom Diary—4 Dimensional, a mixed-methods study by Tom Lormans, Everlien de Graaf, Carlo Leget and Saskia Teunissen in Palliative Care and Social Practice

## Declarations

**Ethics approval and consent to participate:** The study was conducted in compliance with the principles of Good Clinical Practice and in accordance with the principles of the Declaration of Helsinki and the General Data Protection Regulation. As a part of the INZICHT project, the Medical Research Ethics Committee Utrecht confirmed that the Medical Research Involving Human Subjects Act did not apply to this study (19-602/C, 2019). All participants have consented to participate and have been adequately informed about the research they were participating in.

**Consent for publication:** By consenting to participate in this research, all participants consented to the publication of the results of this research.

**Availability of data and materials:** Primary data used in this research can be acquired by contacting the second author of this paper.
